# Evaluation of Stem Cell-Derived Red Blood Cells as a Transfusion Product Using a Novel Animal Model

**DOI:** 10.1371/journal.pone.0166657

**Published:** 2016-12-13

**Authors:** Sandeep N. Shah, Monique P. Gelderman, Emily M. A. Lewis, John Farrel, Francine Wood, Michael Brad Strader, Abdu I. Alayash, Jaroslav G. Vostal

**Affiliations:** 1 Laboratory of Cellular Hematology, Division of Hematology Research and Review, Office of Blood Research and Review, Center for Biologics Evaluation and Research, Food and Drug Administration, Silver Spring, Maryland, United States of America; 2 Laboratory of Biochemistry and Vascular Biology, Division of Hematology Research and Review, Office of Blood Research and Review, Center for Biologics Evaluation and Research, Food and Drug Administration, Silver Spring, Maryland, United States of America; Southern Illinois University School of Medicine, UNITED STATES

## Abstract

Reliance on volunteer blood donors can lead to transfusion product shortages, and current liquid storage of red blood cells (RBCs) is associated with biochemical changes over time, known as ‘the storage lesion’. Thus, there is a need for alternative sources of transfusable RBCs to supplement conventional blood donations. Extracorporeal production of stem cell-derived RBCs (stemRBCs) is a potential and yet untapped source of fresh, transfusable RBCs. A number of groups have attempted RBC differentiation from CD34^+^ cells. However, it is still unclear whether these stemRBCs could eventually be effective substitutes for traditional RBCs due to potential differences in oxygen carrying capacity, viability, deformability, and other critical parameters. We have generated *ex vivo* stemRBCs from primary human cord blood CD34^+^ cells and compared them to donor-derived RBCs based on a number of *in vitro* parameters. *In vivo*, we assessed stemRBC circulation kinetics in an animal model of transfusion and oxygen delivery in a mouse model of exercise performance. Our novel, chronically anemic, SCID mouse model can evaluate the potential of stemRBCs to deliver oxygen to tissues (muscle) under resting and exercise-induced hypoxic conditions. Based on our data, stem cell-derived RBCs have a similar biochemical profile compared to donor-derived RBCs. While certain key differences remain between donor-derived RBCs and stemRBCs, the ability of stemRBCs to deliver oxygen in a living organism provides support for further development as a transfusion product.

## Introduction

Approximately 15 million units of red blood cells are collected in the United States on a yearly basis, and all but 5% are transfused [1]. Transient shortages of blood for transfusion may occur when demand increases, such as in times of natural disasters. Additionally, the need for transfusion products is expected to rise due to increased healthcare demands of the aging US population. While the frequency of disease transmission from transfusion products in the US is low due to rigorous testing and blood donor screening, many developing nations lack the infrastructure to achieve this level of safety [2]. Furthermore, emerging blood borne diseases that lack an appropriate testing method have the potential to become a global threat. Patients who require chronic transfusions may be at risk of transfusion reactions such as developing alloantibodies, which further limits the availability of compatible blood products [3]. These observations indicate that there is room for improvement in the quality and availability of red blood cells for transfusion.

*Ex vivo* expansion and differentiation of red blood cells (RBCs) from stem cells have been intensely studied as a possible means to supplement conventional blood donations [4–7]. A stem cell-derived RBC (stemRBCs) product has the potential to be pathogen free, universally matched to all recipients and be in abundant supply [7]. A number of groups have developed protocols to stimulate differentiation of induced pluripotent stem cells or hematopoietic stem cells to mature into enucleated erythrocytes. While RBCs produced using these methods show much promise, the methods have generally suffered from low cell expansion rates or low enucleation frequency [6]. Due to recent refinements of the techniques, stemRBCs with similar morphology and hemoglobin function compared to donor-derived RBCs have been produced (for review, see [6, 7]). As a proof of concept of their clinical significance, Giarratana *et al*. were able to show that stemRBCs generated *in vitro* could survive in a human subject, with a half-life of approximately 26 days [8].

We analyzed a comprehensive set of parameters to determine the comparability and efficacy of stemRBCs produced by currently established methods vs. donor-derived RBCs. We also developed a novel exercise-induced oxygen debt recovery test to determine in vivo the oxygen delivery potential of stemRBCs. Based on these tests, we determined that the stemRBCs were functional in terms of oxygen delivery in an animal model of transfusion.

## Materials and Methods

### Directed differentiation of CD34^+^ cells to stemRBCs

StemRBCs were derived from cord blood CD34^+^ cells (Stem Cell Technologies, Vancouver, BC, Canada) using a protocol described in Griffiths *et al*., 2012 [9] and slightly modified as follows: Step 1 (day -4–0): Cord blood CD34^+^ cells were maintained in SFEM II + cc100 (1:100 dilution containing Flt3 ligand, SCF, IL-3, IL-6; Stem Cell Technologies); Step 2 (day 0–8): Cells were expanded in base media–IMDM/Glutamax (Life Technologies, Carlsbad, CA), 3% human AB serum (Gemini Bio Products, Sacramento, CA), 2% FBS (FBS, ATCC, Manassas, VA), 10 μg/mL insulin (Sigma Aldrich, St. Louis, MO), 3 U/mL heparin (Sigma Aldrich), and 200 μg/mL holo-transferrin (BBI Solutions, Madison, WI)–supplemented with 1 ng/mL IL-3 (Miltenyi Biotec, San Diego, CA), 10 ng/mL SCF (Miltenyi Biotec) and 3 U/mL EPO (ProSpec Bio, Rehovot, Israel); Step 3 (day 8–11): Cells were placed in base media containing 10 ng/mL SCF, 3 U/mL EPO, and a total of 1 mg/mL holo-transferrin to promote further expansion and maturation; Step 4 (Day 11–18+): Maturation to enucleated stemRBCs was induced by incubating cells in base media supplemented with only 3 U/mL EPO and a total of 1 mg/mL holo-transferrin. Media were changed every 3–4 days. Where indicated, cells were filtered through a non-woven fabric filter [10] (Antoshin, Singapore) to reduce nucleated cells. Briefly, the filter was washed with PBS, and cells diluted to 5–10x10^7^/mL PBS were slowly passed through. The resulting cell suspension was washed in PBS and concentrated to a final volume of 4x10^9^/mL.

### Biochemical parameter analysis

2,3-diphosphoglycerate (2,3-DPG), glucose-6-phosphate dehydrogenase (G6PDH), and adenosine triphosphate (ATP) were analyzed using commercially available assays (from Roche Diagnostics, Indianapolis, Indiana; Trinity Biotech, Bray, Ireland; and Sigma Aldrich, respectively). All cells were washed twice with phosphate buffered saline (PBS, Life Technologies) prior to performing the assays. Additionally, peripheral blood from healthy blood donors (obtained from the Department of Transfusion Medicine, National Institutes of Health, Bethesda, MD) was kept on a rocker at room temperature for 1 day and subsequently analyzed alongside each assay (henceforth referred to as “control RBCs”). Donors voluntarily consented to donate blood with full knowledge that their blood would be used for research. The collection and usage of whole blood for research was performed with the approval of the IRBs from the NIH Clinical Center, Bethesda, MD and FDA Center for Biologics Evaluation and Research (CBER) committee on Research Involving Human Subjects Committee (RIHSC), respectively. As previously described [11], the 2,3-DPG content was measured using 100 μL of cells at a concentration of 4x10^9^/mL and added to 500 μl ice cold 0.6 M perchloric acid (Sigma Aldrich). The suspension was mixed and centrifuged, and 400 μL of the supernatant was collected and neutralized with 50 μL of 2.5 M potassium carbonate (Acros Organics, Geel, Belgium). The neutralized solution was incubated on ice for 1 h, and 100 μL was used for each assay according to the manufacturer’s instructions. Absorbance values were determined using an HP 8453 spectrophotometer (Agilent, Palo Alto, CA). To determine G6PDH activity, 10 μL of cells at a concentration of 4x10^9^/mL were processed using a G6PDH quantitative kit according to the manufacturer’s instructions, and absorbance values were also determined using the spectrophotometer. To measure ATP activity, 4x10^4^ cells were assayed per reaction using an Adenosine 5′-triphosphate (ATP) bioluminescent somatic cell assay kit according to the manufacturer’s instructions. Bioluminescence was determined using a Biotek Synergy-4 plate reader (Winooski, Vermont). An ATP standard (Sigma Aldrich) was used as an internal reference to determine ATP concentration. Hemoglobin analysis was performed using a Hemoglobin Assay kit (Stanbio Laboratories, Boerne, TX).

### Osmotic fragility analysis

Osmotic fragility of red blood cells was determined by exposing cells to increasing dilutions of saline. Briefly, 2 x10^7^ cells were added to PBS solutions from 0% to 0.9% saline and incubated for 30 minutes as previously described [12]. After centrifugation, hemoglobin release, which directly corresponds to the number of lysed cells, was assessed by colorimetric assay by determining the absorbance of the supernatants at 540 nm using the Biotek Synergy-4 plate reader. Control RBCs were also assayed in each osmotic fragility test (according to the original method of Ernest Beutler [13, 14]).

### Oxygen equilibrium curves (OECs)

Oxygen equilibrium binding studies were carried out using a Hemox Analyzer (TCS Scientific, New Hope, PA) using the Hemox buffer (135 mM NaCl, 5 mM KCl, and 30 mM Tes; pH 7.4 at 37°C) provided by the manufacturer. Cells were washed twice and resuspended in PBS at 4 x10^9^ cells/mL. OECs were generated by calculating RBC oxygen saturation in the presence of increasing oxygen partial pressure as previously described [11]. A computer-based analysis of oxygen binding curves for the RBCs was performed, yielding P_50_ and *n*_50_ for oxygen binding.

### Mass spectrometric analysis

Prior to MS analysis, the total protein concentration of all stemRBC lysates (and RBC control) was determined using a standard BCA assay. Afterward, samples were tryptically digested, desalted, and analyzed by mass spectrometry as previously described [15]. Briefly, 1 ug of tryptic peptides representing each sample were analyzed (three technical replicates for each sample) by reverse phase liquid chromatography mass spectrometry (RP LC/MS/MS) using an Easy nLC II Proxeon nanoflow HPLC system coupled online to a Q-Exactive Orbitrap mass spectrometer (Thermo Scientific). Data were acquired using a top10 method (for 120 minutes), dynamically choosing the most abundant precursors (scanned at 400–2000 m/z) from the survey scans for HCD fragmentation. Semi-quantitative spectral counting data used for determining hemoglobin subunit ratios (over several time points in the red blood cell population) were obtained using the Mascot search engine and Scaffold 4.40 software as previously described (15). All other quantitative data processing and visualization was performed using Protalizer DDA software (Vulcan Analytical, Birmingham, AL) version 1.1.2418 [16]. Peptide and protein identifications were made using the X! Tandem Piledriver search engine version 2015.04.01.1 [17] against the forward and reversed human Swiss-Prot database containing 20,202 sequences (not including decoys) downloaded July 10^th^ 2016 with a 20 ppm parent and fragment ion mass tolerance. MS1 and MS2 peaks were calibrated by the median error values for all peptide precursor and fragment ions within each 25 *m/z* mass range bin across the entire scan range. The calibrated spectra were then researched with a more stringent tolerance of 10 ppm parent and 15 ppm fragment ion mass tolerance. Potential modifications searched included oxidation of M residues, deamidation of Q and N residues, pyro-glutamic acid at N-terminal E and Q residues, and N-terminal acetylation. Carbamidomethylation of cysteine residues was searched as a static modification. Peptides with up to 1 trypsin miscleavages were included in the analysis. Only peptides detected at a 1% protein false discovery rate (FDR) were reported by the algorithm based on a target-decoy search strategy comparing the number of decoy reversed identifications to those made in the actual human database. Quantification by MS1 precursor AUC intensities was performed as described previously [16]. To calculate protein ratios within sample types, intensity-based absolute quantitation (iBAQ) was used by dividing the total intensity values of hemoglobin by the intensity of total peptides 6–30mers in length [18]. For calculating protein-level relative abundance across the biological conditions compared, peptides detected in each sample were used whenever possible. Median peptide relative abundance and P-Values from an unpaired t-test were reported for all the peptides used to quantify a target protein in the protein quant summary.

### Mouse models

Animal protocols were approved by the FDA CBER Institutional Animal Care and Use Committee, and all experimental procedures were performed in compliance with the National Institutes of Health guidelines on the use of experimental animals. SCID mice, 6–8 weeks of age, were purchased from NCI/DCT and acclimated in an on-site pathogen-free facility for a minimum of 1 week. Heterozygous Hbb^th3/+^ mice on a C57BL/6J-B6 background were bred in-house (henceforth referred to as “anemic C57BL/6J mice”) [19]. We created SCID Hbb^th3/+^ mice in-house 2-years prior to commencing this current study (henceforth referred to as “anemic SCID mice”). These SCID mice have a mild to moderate anemia due to β-thalassemia. Experimental mouse cohorts were age-matched. Murine RBCs from WT C57BL/6J mice and stemRBCs were labeled with carboxyfluorescein succinimidyl ester (CFSE; Abcam, Cambridge, MA) and washed twice in PBS. The anemic SCID mice were infused with either CFSE-labeled murine RBCs, filtered stemRBCs (10^9^ cells in 300 μL of PBS), or 300 μL saline by intravenous injection (tail vein). Whole blood was collected in heparinized capillary tubes (Fisher Scientific, Waltham, MA) using the tail vein nick technique at predetermined time points, starting at 5 min post transfusion. Lactate levels were also measured at these time points using a Lactate-plus apparatus (Nova Biomedical, Waltham, MA). Thirty minutes post-transfusion, mice were subjected to a 15 minute swim exercise test [20] in a cylindrical glass tank (20 cm height x 30 cm diameter) which was filled with 9 L of water at 26°C containing a small amount of soap to reduce surface tension and discourage floating behavior [21]. In addition, the tank was filled ~13 cm from the bottom to make sure that the mouse could not reach the bottom during the test. Lactate levels were measured immediately before and after the swim and then at 15-minute intervals up to 2 hours.

### Flow cytometry

Flow cytometry was used to characterize the cells at various stages of differentiation. The cells were incubated with FITC- or PE-conjugated monoclonal antibodies against CD34, CD36, CD44, CD47, CD71, and glycophorin-A (GPA) for 20 min. Monoclonal antibodies and appropriate isotype controls were purchased from Becton Dickinson and Invitrogen (Carlsbad, CA). Cells were washed twice and analyzed using a FACSCalibur (Becton Dickinson, San Jose, CA) equipped with CellQuestPro software. To determine recovery of transfused RBCs, mouse whole blood samples containing either CFSE-labeled control RBCs or stemRBCs were washed twice and resuspended in 500 μL PBS/0.1% BSA for flow cytometric analysis [12].

### Microscopy

Cell size, count and viability were determined using a Nexcelom Cellometer Auto X4 (Lawrence, MA) with Acridine Orange/Propidium Iodide staining solution (AO/PI, Nexcelom Bioscience). After day 14, cell count and viability were determined using a hemacytometer and trypan blue solution (Mediatech, Inc., Manassas, VA). All brightfield cell images were captured using an Olympus CK40 microscope (Center Valley, PA) with QCapture Pro v6.0 software (QImaging, Surrey, Canada), and images of stained slides were captured using an Olympus IX73 microscope with cellSens Dimension v1.12 software (Olympus). To measure enucleation frequency, cells were centrifuged onto slides using a Cytospin 4 (Thermo Scientific, Waltham, MA), fixed, stained with Wright-Giemsa, and visualized under 40x magnification. Three independent observers each counted 3 random fields of 100 cells, in a blinded fashion, to obtain an average value. Enucleation frequency was calculated by dividing the number of enucleated cells by the total number of cells.

## Results

### Cell proliferation and differentiation

**Fig 1:**
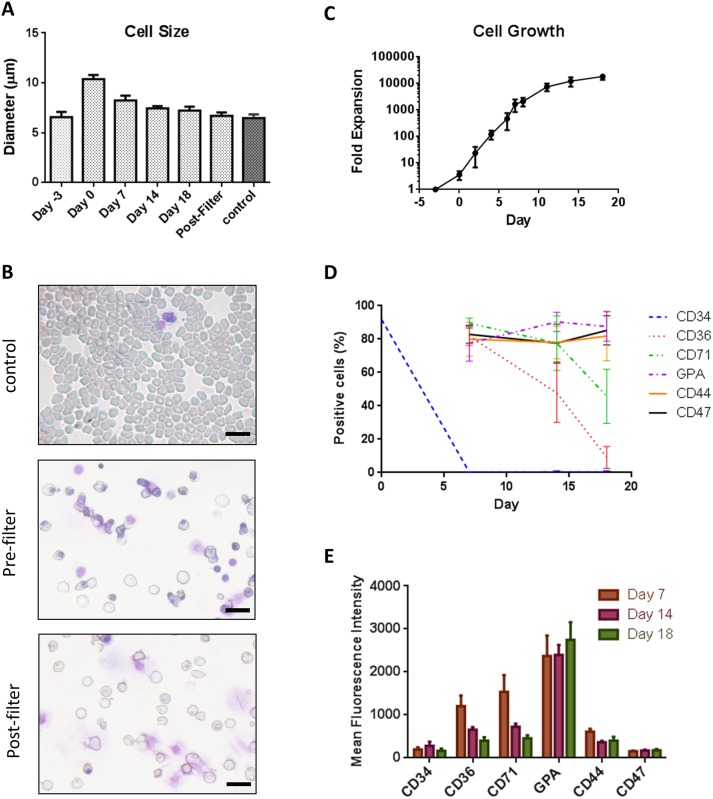
Characterization of stemRBCs. (A) Cell diameters of stemRBCs (n = 10) derived from cord blood isolated CD34^+^ cells during differentiation from day 0 to day 18 in culture compared to human control RBCs and filtered cells on day 18. (B) Cell morphology of adult human blood (control) from cytospins for comparison with pre- (NF) and post-filter (F) day 18 stemRBCs. Scale bar represents 20 μm. (C) Total number of viable, non-adherent cells were determined and plotted as a mean ± SE of 4 cultures. (D) Frequency of cells expressing several erythroid markers as determined by flow cytometry over an 18 day culture period (mean ± SE) and (E) mean ± SE fluorescence intensities (MFIs) showing expression patterns of these markers during differentiation (n = 8).

Primary human cord blood CD34^+^ cells were cultured using an 18-day, 4-step protocol to generate stemRBCs. The starting average cell size was ~6.6 μm for CD34+ (day -3), which increased to 10 μm on day 0 and then gradually decreased to 7.2 μm by day 18 (Fig 1A). The mean corpuscular volume (MCV) of day 18 (nonfiltered and post-filtered) cells was 134 and 119 fL, respectively, which was higher than reference volumes listed on MedlinePlus [22] (Table 1). On day 18, cell cultures were still heterogeneous in terms of cell size and differentiation state. Additionally, a number of expelled nuclei surrounded by a plasma membrane (which resembled pyrenocytes [23]) could be observed (Fig 1B). These pyrenocytes were very adhesive and were mostly present in large clusters. The majority of larger, more immature cells as well as the pyrenocytes were removed by filtration on day 18, yielding a cell population with an average cell size of 6.7 μm, which was comparable to control RBCs (6.5 μm) (Fig 1A). The cell expansion between day -3 and 18 was 18,700-fold (Fig 1C). Cell division primarily occurred between days -3 and 11 when IL-3 and/or SCF were present. The viability of the cells was greater than 90% for the duration of the culture period. Based on Wright-Giemsa staining, 53.4% of the cells were enucleated on day 18, with no further enucleation thereafter. Following removal of premature cells and pyrenocytes by filtration, 82% of the remaining cells were enucleated.

**Table 1:** Mean corpuscular volumes (MCV) of filtered (F) and non-filtered (NF) stemRBC and reference RBC values. MCV was determined on Day 18 stemRBC using a Cell Dyne 3700 analyzer, (Abbott Diagnostics, IL).

MCV	
**Stem RBC (F)**	134 fL
**Stem RBC (NF)**	119 fL
**Reference**	80–96 fL

### Characterization of cells using flow cytometry

During each step of the differentiation process, the cells were labeled with antibodies for various markers of erythrocyte maturation (Fig 1D and 1E). At step 1, we confirmed that the starting cell population was mostly CD34-positive (87–96%) but, by day 8, less than 1% of cells expressed CD34 (Fig 1D). The frequency of cells expressing CD36, an early marker of erythroid differentiation, decreased from 85% during step 2, to 64% during step 3, and finally to 15% during step 4 (Fig 1D). The frequency of cells expressing CD44, which is expressed on hematopoietic cells, remained constant at 85–87% throughout the culture process (Fig 1D). The percentage of cells that were CD47 positive, expressed on all hematopoietic cells and important for protection against clearance by macrophages, also remained relatively constant (Fig 1D). The frequency of cells expressing CD71, a marker of immature RBCs, such as reticulocytes, decreased from 96% (step 2) to 90% (step 3) to 45% (step 4) (Fig 1D). The mean cell surface expression of CD71 also decreased during differentiation (Fig 1E). Lastly, the frequency of cells expressing CD235a (GPA), a marker of mature RBCs, increased from 65% (step 2), to 80% (step 3), and finally to 92% (step 4) (Fig 1D). Following removal of immature cells by filtration, the frequency of CD71-positive cells remained the same and GPA-positive cells increased to 97%. These frequencies were comparable with those of control adult blood cells (CD44, 96%; CD47, 81%; CD71, <1%; GPA, 95%).

### Hemoglobin expression

**Fig 2:**
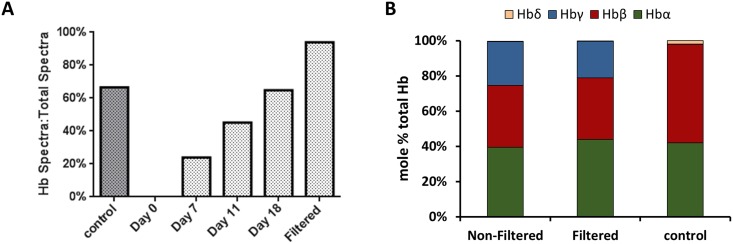
Proteomic analysis of hemoglobin expression. (A) Ratio of spectra (%) matched specifically to Hb tryptic peptides (spectral count) over total matched spectra during erythropoietic differentiation of cord blood-derived CD34^+^ cells along with human control RBCs and filtered cells on day 18. (B) Intensity based absolute quantification (iBAQ) was used to calculate the mole % ratio of peptides unique to α, β and γ Hb subunits over all identified peptides of filtered (F) and non-filtered (NF) cells on day 18 of culture along with human control RBCs. Other Hb subunits were found but were <0.5% of total Hb and are thus not shown on the graph. Standard deviations for α, β, γ, and δ are as follows: (NF): 3.4%, 2.8%, 3.0%, and 0.05%; (F): 4.2%, 9.7%, 5.8, and 0.23%; (control) 6.8%, 7.0%, 0.05%, and 0.36%, respectively.

The total hemoglobin expression of stemRBCs at day 18 ranged from 12 to 23 g/dL, with no difference after filtration (n = 3 and n = 5 for non-filtered and filtered cells, respectively (S1 Fig). A number of labs have reported the persistence of fetal hemoglobin (HbF) in RBCs differentiated from cord blood [24–26]. Mass spectrometry was performed to determine the ratios of various hemoglobin subunits in the red blood cell population. Using spectral counting as a semi-quantitative metric, the ratio of spectra matched specifically to Hb peptides over all matched spectra increased from 24% during step 1, to 65% during step 4, indicating increased accumulation of Hb as cells progressed through the differentiation stages. After filtration, Hb-specific spectra increased to 94%, possibly due to removal of potentially low Hb-expressing progenitor cells (Fig 2A). To determine the actual ratios of individual Hb subunits, quantitative mass spectrometry using ion current measurements was used to determine the amount of peptides corresponding to specific Hb subunits relative to all identified peptides. At the final stage of differentiation, 25%, 21%, and 0.2% of Hb subunits were fetal type (Hbγ) in non-filtered and filtered stemRBCs and control, respectively. As expected, control RBCs contained mostly adult type hemoglobin (Hbα 42%, Hbβ 56%, Hb) (Fig 2B). Filteration significantly reduced cells expressing fetal (Hbγ) and embryonic type (Hbζ, Hbε) hemoglobin subunits (Table 2). This is reflected by the 7 fold decrease (Hbγ) in non-filtered versus filtered samples. Based on mean corpuscular hemoglobin (MCH) and mean corpuscular hemoglobin concentration (MCHC) values, stemRBCs had similar concentrations of hemoglobin per cell volume compared to adult human RBCs (reference values), although stemRBCs contained more hemoglobin per cell (Table 3), since they are larger than normal RBCs.

**Table 2:** Fold change comparison of hemoglobin subunits between stemRBC and control RBC. Non-filtered and filtered stemRBC on day 18 of culture along with human control RBCs. Mean, (n = 3).

	Non-Filtered	Filtered	Control	P value		
				Non-filtered vs. Filtered	Non-filtered vs. Control	Filtered vs. Control
**Hbα**	1.18	1.11	1	1.85E-02	1.52E-02	7.00E-04
**Hbβ**	1	1.08	3.97	4.63E-02	2.74E-08	8.16E-07
**Hbγ-1**	258.1	37.07	1	2.00E-04	1.00E-04	2.50E-05
**Hbγ-2**	100.56	34.36	1	1.00E-04	4.71E-05	1.00E-04
**Hbζ**	182.32	11.73	1	2.10E-06	7.55E-06	7.60E-03
**Hbδ**	1.2	1	16.96	2.36E-01	1.74E-10	1.82E-10
**Hbε**	198.52	49.6	1	1.04E-01	1.00E-04	1.74E-02

**Table 3:** MCH and MCHC values of stemRBC and control RBC. Filtered (F) stemRBCs (Day 18) along with reference values for control RBCs obtained from MedlinePlus. Mean, (n = 3).

	MHC	MCHC
**stemRBC (F)**	49 pg	37 g/dl
**stemRBC (NF)**	n.d.	n.d.
**Reference**	27–33 pg	33–36 pg

### Measurement of oxygen binding and structural parameters

**Fig 3:**
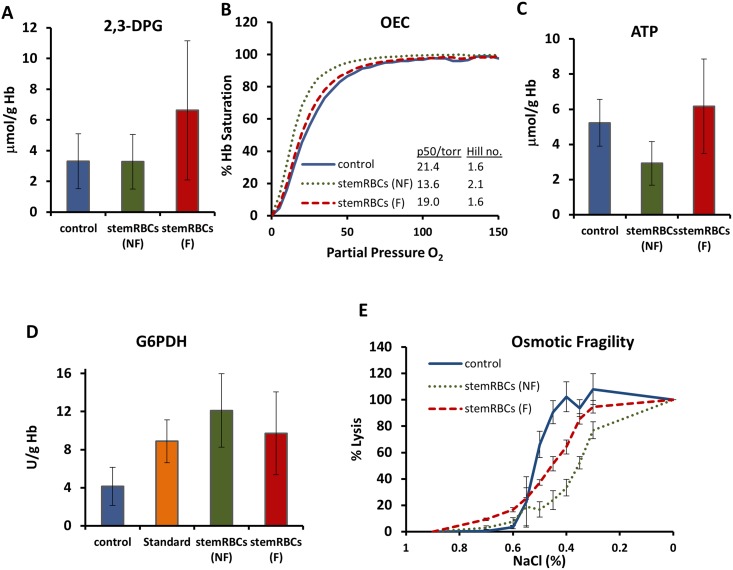
Analysis of biochemical and membrane property parameters. (A) 2,3-DPG levels in human control RBCs, as well as non-filtered (NF) and filtered (F) stemRBCs harvested on day 18 of culture (n = 3)(mean ± SD). (B) Oxygen binding curves showing filtered (F) and non-filtered (NF) stemRBCs harvested on day 18 of culture and control RBCs (n = 3 each) (mean ± SD). (C) ATP and (D) G6PDH analysis of day 18 non-filtered and filtered stemRBCs (n = 3–5) as well as control and G6PDH standard (n = 8) (mean ± SD). (E) Osmotic fragility of filtered and nonfiltered stemRBCs harvested on day 18 was assessed by exposing them to decreasing amounts of saline and determining the amount of released hemoglobin by colorimetric assay (control RBCs were assessed simultaneously for comparison). Error bars show standard deviation.

Oxygen release by hemoglobin is regulated by intracellular 2,3-DPG levels; higher 2,3-DPG concentrations decrease oxygen affinity for hemoglobin and increase oxygen availability to tissues. We observed similar 2,3-DPG levels in non-filtered (NF) and filtered (F) stemRBCs compared to control RBCs (NF: 3.3 μmol/g Hb; F: 6.6 μmol/g Hb; control: 3.3 μmol/g Hb; Fig 3A). As the stemRBCs contained a high amount of fetal Hb (25% of total Hb vs. 8% in control), which has a higher affinity for oxygen than adult Hb, we sought to determine the oxygen binding potential of the stemRBCs. Oxygen equilibrium curves (OECs) were generated using a Hemox Analyzer to measure oxygen saturation of Hb. Compared to control RBCs, the OECs of filtered and non-filtered stemRBCs were left-shifted (P_50_ for control is 21.4 vs. 13.6 torr for non-filtered stemRBCs and 19.0 torr for filtered stemRBCs; Fig 3B), confirming their higher affinity for oxygen.

ATP levels can be used to assess RBC metabolic state. ATP concentrations have a profound influence on the shape and deformability of the RBC membrane [27, 28]. Furthermore, low ATP levels can increase microvascular adhesion of RBCs, causing regional decreases in blood flow or even vaso-occlusion [29]. The mean ATP level of the filtered stemRBCs was 6.2 μmol/g Hb (2.9 μmol/g Hb for non-filtered cells), close to control RBC levels of 5.2 μmol/g Hb (Fig 3C).

Next, we measured the levels of G-6-PDH in our stemRBCs and control RBCs. Reactions involving G-6-PDH produce compounds that protect cells from damage by reactive oxygen species [30]. Typical G-6-PDH levels in human blood range from 4.6–13.5 U/g Hb [31]. G-6-PDH values in the non-filtered and filtered stemRBCs, control RBCs and in a G-6-PDH standard control were all within this range (12.1, 9.7, 4.1, and 8.9 U/g Hb, respectively; Fig 3D).

The osmotic fragility of RBCs determines their susceptibility to hemolysis, and can be used as an index of membrane stability and composition [32]. StemRBCs were more resistant to osmotic stress than control RBCs (Fig 3E). A 50% lysis of control RBCs occurred at 0.45% saline compared to 0.35% saline for non-filtered stemRBCs. The durability of stemRBCs may come from the heterogeneity of the population, as nucleated cells may be less susceptible to hemolysis. Following filtration, which generated a cell population with 82% enucleated red cells, lysis of 50% of stemRBCs occurred at 0.45% saline, similar to the control. However, the stemRBC fragility curve was less sigmoidal than for control RBCs. This may reflect the uniform young age of all of the stemRBCs as compared to control RBCs, which will have a range of young and old red cells due to unsynchronized release from the bone marrow.

### Functional tests of stemRBCs in an animal model of transfusion

**Fig 4:**
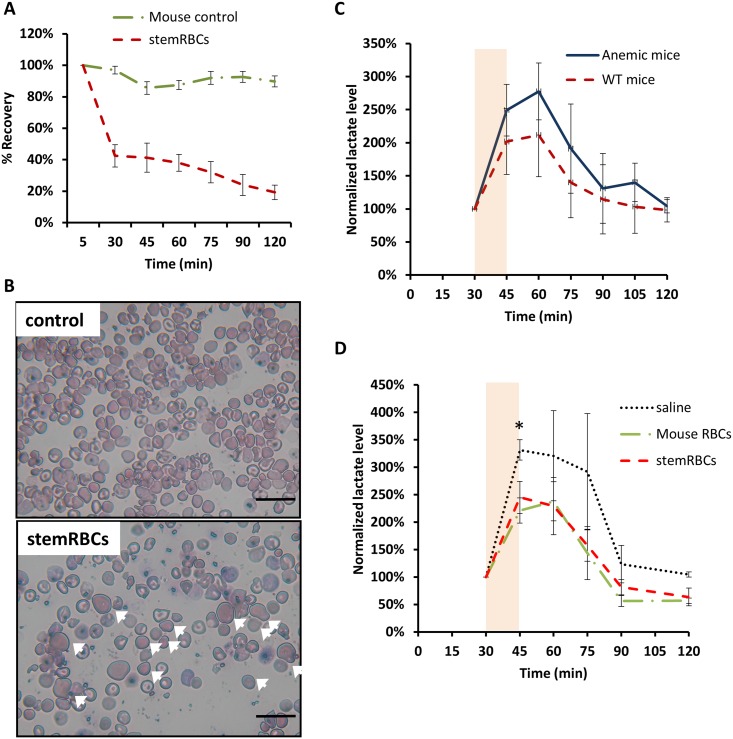
Functional analysis of stemRBCs *in vivo*. (A) In vivo recovery of day 18 stemRBCs and WT mouse control RBCs (n = 4–6 per group) in anemic SCID mice. CFSE-labeled cells were infused by i.v. injection (tail vein). Mouse whole blood was collected at the indicated times and analyzed by flow cytometry. Recovery was set to 100% at the 5 min time-point. (B) Representative images of mouse whole blood collected at 5 minutes post-injection of murine control RBCs or stemRBCs (arrows). Scale bar: 20 μm. (C) The animal model of oxygen function was validated by subjecting anemic C57BL/6J (Hbb^th3/+^) and wildtype C57BL/6J mice (n = 3 per group) to a 15 min swim (shown as an orange rectangle) in 26°C water and ambient air. Lactate levels were measured pre- and post-swim, and every 15 min thereafter. Oxygen deficit due to exercise is reported in mice through a corresponding spike in lactate level. (D) stemRBCs, along with controls (saline and wild-type mouse RBCs; n = 3–5 per group) were introduced into C57BL/6J Hbb^th3/+^ mice. After 30 min, lactate was measured, and mice were subjected to a 15 min swim. Lactate was measured post-swim and every 15 min as indicated. Data points are means and error bars show SEM. (* = p< 0.05).

To determine circulation kinetics, filtered stemRBCs and murine RBCs from WT C57BL/6J mice were labeled with CFSE dye and introduced into anemic SCID mice by i.v. injection. Mouse whole blood was collected at predetermined time points starting at 5 min to 2 h post-infusion to determine the presence of the transfused cells in circulation. For stemRBCs, 50–60% of the transfused cells were cleared by 30 min, while 97% of mouse RBCs were still present at this time-point. At 2 h, 20% of stemRBCs and 90% of mouse RBCs persisted in circulation (Fig 4A).

To measure the ability of stemRBCs to deliver oxygen to tissues, we developed a novel exercise endurance test using plasma lactate levels as an output for oxygen debt of working muscles. In this test, anemic SCID mice were transfused with stemRBCs or various controls; 30 min after transfusion, mice were forced to swim in a pool of room temperature water for 15 min. Baseline lactate levels were determined prior to the swim, immediately post-swim and then predetermined time points until levels returned to baseline. Lactate levels were normalized to the baseline level for each animal and plotted against time. As a proof-of-principle, we subjected non-transfused anemic (Hbb^th3/+^) and non-anemic C57BL/6J (WT) mice to the same swim test. As shown in Fig 4C, there was a higher peak of lactate post exercise and greater total lactate accumulation in anemic mice during the swim compared to WT mice. This indicates that the test can effectively be used to confirm the greater oxygen availability to muscle tissue in WT mice. Next, anemic SCID mice were transfused with stemRBCs, as well as the following controls: RBCs from WT murine blood (Mouse RBCs; positive control) and saline (negative control) (Fig 4D). Anemic mice that received transfusions of WT mouse RBCs had comparable lactate levels observed in Fig 4C for WT non-transfused mice (respectively, 220% vs. 202% lactate increase immediately after swim, and 238% and 212% lactate increase 15 min after swim). This indicates that the mouse cells could successfully rescue the oxygen deficit in anemic animals. Control mouse RBC and stemRBC-transfused animals had a similar lactate spike following the swim, and both infusions of cells produced lactate levels lower than saline. These observations indicate that in this animal model stemRBCs delivered oxygen as well as mouse RBCs, and both performed better than saline alone. (Fig 4A).

## Discussion

Stem cell-derived RBCs are being considered as a potential alternative to donor-derived red cell transfusion products [6, 7, 33, 34]. We characterized biochemical and functional parameters of RBCs derived from cord blood and compared those parameters to RBCs collected from healthy human volunteers. We used a modified culture protocol based on Griffiths et al. [9], which, in our hands, resulted in the highest amount of cell expansion and enucleation vs. other methods, and comparable morphology to normal human erythrocytes [9, 33, 35, 36].

High enucleation efficiency is an important parameter for characterizing stem cell-derived red cells because of the uncertainty whether nucleated red cells would make suitable transfusion products. The enucleation efficiency obtained by our culture was comparable to those by other groups with a co-culture-free system for erythrocyte differentiation starting from cord blood-derived progenitor cells [35–37]. In our study, filtration removed approximately 75% of cells, even though only 53% were nucleated. Thus, culture conditions to improve enucleation or methods to remove nucleated cells need to be optimized. By extending the culture duration, a number of studies showed expansion rates as high as 10^8^ to 10^9^ [38, 39]; however, enucleation rates were either not reported or were much lower than what we observed in our study.

Compared to control RBCs, our stemRBCs were slightly larger in size (Table 1). This is consistent with the observation by Timmons *et al*. who showed that stemRBCs were 40% larger than donor erythrocytes [38]. The stemRBCs had equal concentrations of hemoglobin as adult donor RBCs based on MCHC, although due to their larger size, stemRBCs contained 50% more hemoglobin per cell based on MCH values.

Fetal hemoglobin has a higher oxygen affinity than adult hemoglobin, and its presence can shift oxygen dissociation curves to the left [40]. We observed this on our oxygen dissociation curves, where stemRBCs displayed a slightly left-shifted curve. A study comparing oxygen saturation levels of erythrocytes derived from cord blood and adult peripheral blood CD34^+^ cells found that both cell populations had equally greater oxygen affinity compared to control adult peripheral blood [41], while other groups showed similar oxygen dissociation curves between control blood and stemRBCs from peripheral blood CD34^+^ cells or hESCs [8, 42].

While high fetal hemoglobin is not typical for adult donor-derived RBCs, this may not be problematic for a transfusion product. Hereditary persistence of fetal hemoglobin (HPFH) has been reported in some individuals who have high levels of fetal hemoglobin throughout life (20–35% in heterozygotes and 100% for homozygotes), but are mostly asymptomatic [43–45]. RBCs with high levels of HbF have also shown benefit to individuals with β-globin disorders such as sickle cell disease; for these individuals, HbF interferes with the polymerization of HbS (the defective hemoglobin) and prevents cell damage [46].

A critical issue in the development of a red blood cell transfusion product is the demonstration of its ability to deliver oxygen to tissues. Ultimate validation of this will require a clinical trial, but a pre-clinical determination of the capacity to deliver oxygen in an animal model would be a favorable indication for further product development. One study demonstrated stemRBC circulation in a single human subject, and showed a comparable half-life to control RBCs, validating the feasibility of ex vivo-generated RBCs [8]. A number of animal models have been utilized to test tissue oxygenation with hemoglobin-based oxygen carriers (HBOC) [47]. Some of these studies have measured tissue oxygen tension or oxygen concentration using microelectrodes or phosphorescence, respectively [47]. While these methods have high precision, the former method is invasive, and both can only provide information for a very localized region. Thus, a reliable *in vivo* system is needed to globally assess oxygen delivery and oxygen utilization following blood transfusion. Ideally, this system should involve conscious animals to avoid confounding influences of sedatives on tissue oxygenation.

A number of other studies have utilized forced swimming as a means to determine exercise capacity in mice treated with various compounds [48, 49]. These studies used plasma lactate as a measurable parameter of exercise-induced fatigue (oxygen debt). Plasma lactate levels correlate with increases in anaerobic respiration, which occur when oxygen demand surpasses availability, such as in times of intense exercise [50]. A recent clinical trial of red blood cell efficacy in humans has also utilized plasma lactate levels as an endpoint for oxygen delivery to evaluate differences between fresh and stored blood [51].

To our knowledge, we report here the first study to employ an *in vivo* test to determine the oxygen delivery potential of stemRBCs. In this model, anemic animals produced higher levels of lactate than non-anemic animals in response to the same level of exercise. The lactate levels were reduced if the anemic animals were transfused with mouse whole blood prior to the exercise, indicating that in this model, oxygen delivery capacity was limited by the levels of circulating RBCs capable of binding and releasing oxygen. Transfusing WT mouse RBCs or human stemRBCs into this animal model reduced lactate buildup compared to animals infused with the same volume of saline, thus indicating that the transfused cells did improve the oxygen carrying capacity of blood. Although the clearance of infused stemRBCs was much faster than infused murine cells, exercise-induced oxygen debt was similarly reduced. This may be due to the fact that the stemRBCs contained much more hemoglobin per cell than murine RBCs (49 pg/cell vs. 16 pg/cell) (S2 Fig), and consequently had greater oxygen carrying capacity. Together, our data suggests that stemRBCs can accept oxygen in the lungs and deliver it to working muscle tissues in a mouse model. This mouse model can be used as a rapid pre-clinical test of stemRBC effectiveness prior to future therapeutic applications in humans.

## Supporting Information

S1 Fig Total amount of Hemoglobin in Non-Filtered and Filtered stem RBC. Total amount of hemoglobin (g/dL) in stemRBCs at day 18 with or without filtration (n = 3 non-filtered and n = 5 filtered cells). Mean ± SEM.
(DOC)

S2 Fig Mean corpuscular hemoglobin (MCH) of filtered stem RBCs (F) and murine RBCs. MHC determined for day 18 filtered stemRBCs (n = 3) and murine RBCs (n = 3). Mean ± SEM.
(DOC)
