# Supplementary material for: Evaluation of Stem Cell-Derived Red Blood Cells as a Transfusion Product Using a Novel Animal Model
Source: PLoS One. 2016 Dec 13;11(12):e0166657. doi: 10.1371/journal.pone.0166657 (PMC5154495; doi:10.1371/journal.pone.0166657)
Supplement: Supplementary file 1 [file pone.0166657.s001.doc]

# S1 Fig

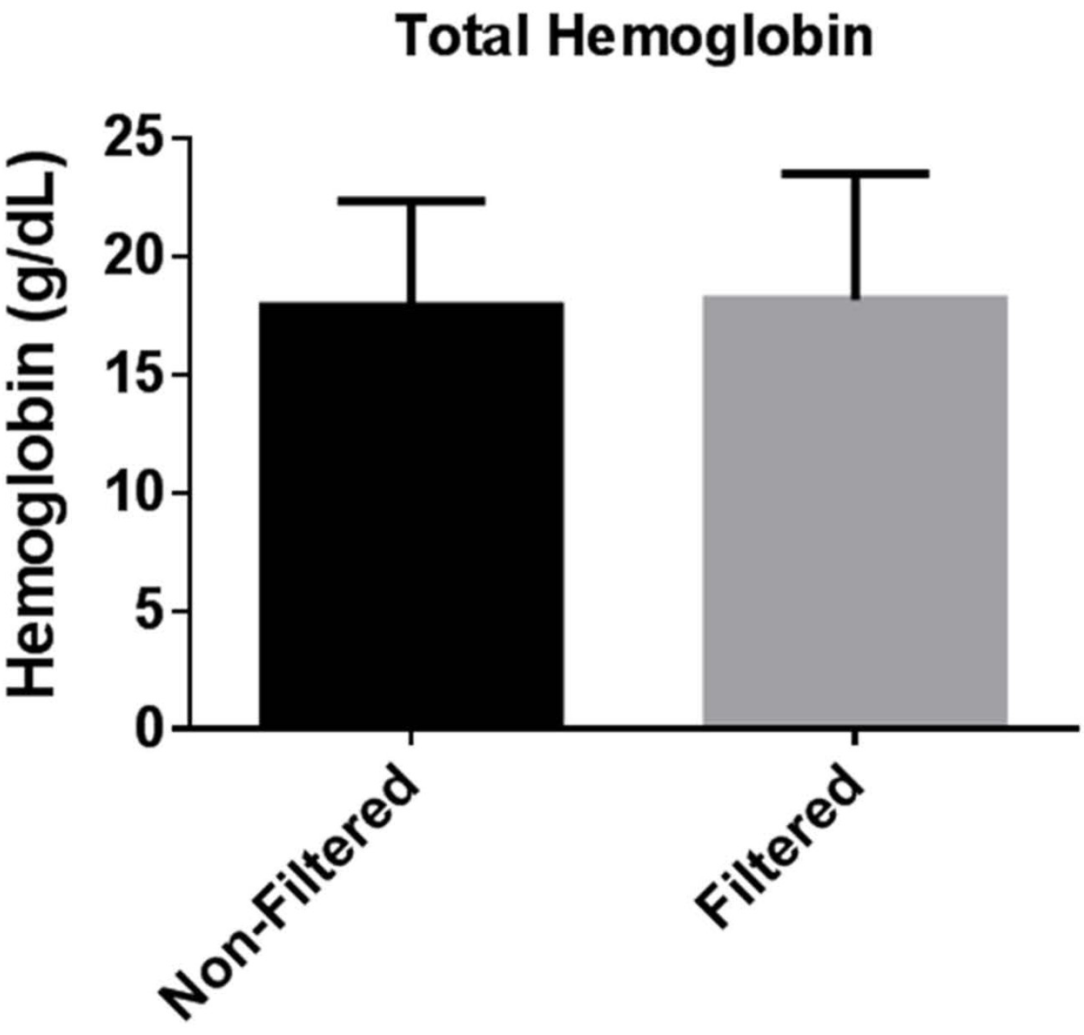

Total Hemoglobin
Hemoglobin (g/dL)
25
20
15
10
5
0
Non-Filtered
Filtered
