# Supplementary material for: Evaluation of Stem Cell-Derived Red Blood Cells as a Transfusion Product Using a Novel Animal Model
Source: PLoS One. 2016 Dec 13;11(12):e0166657. doi: 10.1371/journal.pone.0166657 (PMC5154495; doi:10.1371/journal.pone.0166657)

S2 Fig

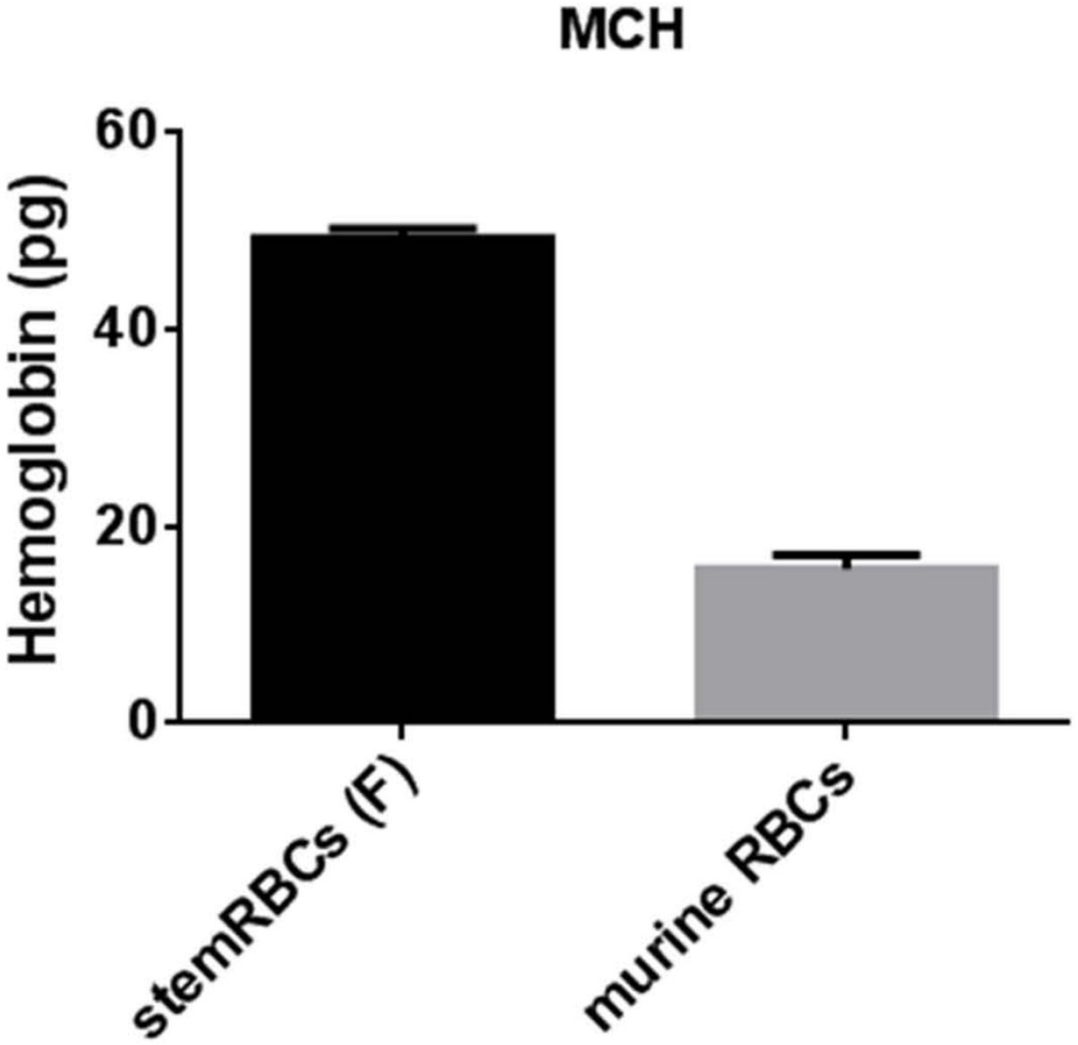

Supplement: Supplementary file 2 [file pone.0166657.s002.doc]
